# Effects of a High-Protein Diet on Cardiometabolic Health, Vascular Function, and Endocannabinoids—A PREVIEW Study

**DOI:** 10.3390/nu12051512

**Published:** 2020-05-22

**Authors:** Lea Tischmann, Mathijs Drummen, Peter J. Joris, Blandine Gatta-Cherifi, Anne Raben, Mikael Fogelholm, Isabelle Matias, Daniela Cota, Ronald P. Mensink, Margriet S. Westerterp-Plantenga, Tanja C. Adam

**Affiliations:** 1 Department of Nutrition and Movement Sciences, Maastricht University Medical Centre, 6200 MD Maastricht, The Netherlands; m.drummen@maastrichtuniversity.nl (M.D.); p.joris@maastrichtuniversity.nl (P.J.J.); r.mensink@maastrichtuniversity.nl (R.P.M.); m.westerterp@maastrichtuniversity.nl (M.S.W.-P.); t.adam@maastrichtuniversity.nl (T.C.A.); 2 NUTRIM School of Nutrition and Translational Research in Metabolism, Maastricht University, 6200 MD Maastricht, The Netherlands; 3 Department of Endocrinology, University Hospital of Bordeaux, F-33607 Pessac, France; blandine.gatta-cherifi@chu-bordeaux.fr; 4 INSERM, Neurocentre Magendie, Physiopathologie de la Plasticité Neuronale, U1215, F-33000 Bordeaux, France; isabelle.matias@inserm.fr (I.M.); daniela.cota@inserm.fr (D.C.); 5 Neurocentre Magendie, Physiopathologie de la Plasticité Neuronale, U1215, University of Bordeaux, F-33000 Bordeaux, France; 6 Department of Nutrition, Exercise and Sports, University of Copenhagen, DK1017 Copenhagen, Denmark; ara@nexs.ku.dk; 7 Department of Food and Environmental Sciences, University of Helsinki, FI-00014 Helsinki, Finland; mikael.fogelholm@helsinki.fi

**Keywords:** protein, cardiometabolic health, vascular function, endocannabinoids

## Abstract

An unfavorable lipid profile and being overweight are known mediators in the development of cardiovascular disease (CVD) risk. The effect of diet, particularly high in protein, remains under discussion. Therefore, this study examines the effects of a high-protein (HP) diet on cardiometabolic health and vascular function (i.e., endothelial function, arterial stiffness, and retinal microvascular structure), and the possible association with plasma endocannabinoids and endocannabinoid-related compounds in overweight participants. Thirty-eight participants (64.5 ± 5.9 (mean ± SD) years; body mass index (BMI) 28.9 ± 4.0 kg/m^2^) were measured for 48 h in a respiration chamber after body-weight maintenance for approximately 34 months following weight reduction. Diets with either a HP (*n* = 20) or moderate protein (MP; *n* = 18) content (25%/45%/30% vs. 15%/55%/30% protein/carbohydrate/fat) were provided in energy balance. Validated markers for cardiometabolic health (i.e., office blood pressure (BP) and serum lipoprotein concentrations) and vascular function (i.e., brachial artery flow-mediated vasodilation, pulse wave analysis and velocity, and retinal microvascular calibers) were measured before and after those 48 h. Additionally, 24 h ambulatory BP, plasma anandamide (AEA), 2-arachidonoylglycerol (2-AG), oleoylethanolamide (OEA), palmitoylethanolamide (PEA), and pregnenolone (PREG) were analyzed throughout the day. Office and ambulatory BP, serum lipoprotein concentrations, and vascular function markers were not different between the groups. Only heart rate (HR) was higher in the HP group. HR was positively associated with OEA, while OEA and PEA were also positively associated with total cholesterol (TC) and low-density lipoprotein (LDL) cholesterol concentrations. Vascular function markers were not associated with endocannabinoids (or endocannabinoid-related substances). In conclusion, the HP diet did not affect cardiometabolic health and vascular function in overweight participants after completing a weight-loss intervention. Furthermore, our data indicate a possible association between OEA and PEA with TC and LDL cholesterol.

## 1. Introduction

Cardiovascular disease (CVD) is the most common cause of death worldwide [1]. Well-known mediators in the development of vascular damage and CVD are an unfavorable serum lipid profile and being overweight [2]. Whilst an unhealthy diet (e.g., high in salt and saturated fat) is known as a risk factor for the development of CVD [2], the effect of macronutrients, in particular the effect of dietary protein in general remains under discussion [3]. Reviews on the relationships between longer-term protein intakes with CVD [4], serum lipids, and blood pressure (BP) [5] are however inconclusive and more research is warranted in this field [6,7,8]. In addition to diet, it has recently been suggested that the endocannabinoid system (ECS) plays a role in the regulation of cardiometabolic health and vascular function. The ECS consists of three components: (1) the cannabinoid receptors (CB1 and CB2), (2) enzymes responsible for the metabolization of ligands, and (3) endogenous ligands. Those ligands are endocannabinoids like anandamide (AEA) and 2-arachidonoylglycerol (2-AG), and endocannabinoid-related compounds like oleoylethanolamide (OEA), palmitoylethanolamide (PEA), and pregnenolone (PREG).

AEA and 2-AG may affect factors directly related to cardiovascular health [9,10,11] including the regulation of blood pressure [11] and may exert beneficial vasoactive properties [10,12]. More specifically, a positive correlation of 2-AG with body mass index (BMI) and body fat percentage, with an unfavorable lipid profile, and a higher glycemic response has been found [9]. For AEA, results are more conflicting, as plasma concentrations vary considerably among studies [9]. Treatment with CB1 antagonists improved plasma lipid profiles [13,14,15], and reduced blood pressure in humans [11], but was also associated with adverse events including psychological problems [16]. Data on the impact of diet on the ECS system are limited, but it may be affected by fat intake [17]. Recently, however, we found a higher dietary protein intake had an enhancing effect on plasma 2-AG concentrations [18].

Currently, human intervention data on the role of the ECS in protein-induced effects on cardiometabolic health and vascular function are limited. Related to our findings of an increasing effect of protein intake on plasma 2-AG concentrations [18], we propose that the ECS is a possible route of action by which dietary protein affects cardiometabolic risk and vascular function markers. Therefore, this study aims to assess the effects of a high-protein (HP) versus a moderate-protein (MP) diet in overweight participants on validated markers for cardiometabolic health and vascular function. As a secondary objective, this study examines a possible association of those markers for cardiometabolic health and vascular function with circulating endocannabinoids and related compounds concentrations. This study was designed as a substudy of the PREVIEW intervention study [19].

## 2. Materials and Methods

The study was performed according to the Declaration of Helsinki and approved by the Maastricht University Medical Centre’s Medical Research Ethics Committee (METC). The PREVIEW study was registered on 29 January 2013 at ClinicalTrials.gov (NCT01777893). Written informed consent for participation was collected from all participants prior to the study. The substudy was performed from February 2017 until February 2018 in Maastricht, The Netherlands.

### 2.1. Study Design

The present study was set up as a substudy to PREVIEW, a study assessing the prevention of diabetes through lifestyle intervention and population studies in Europe and around the world. The PREVIEW study was funded through the European Union (EU) 7th Framework Program (grant agreement no. 31205) [19]. Detailed information on the PREVIEW intervention and on the substudy has been published [18,19]. In brief, the PREVIEW intervention started with 8 weeks of weight loss, followed by 34 months of a randomized intervention with either a moderate-protein (MP), moderate-glycemic index (GI) or a high-protein (HP), low-GI diet, combined with either moderate- or high-intensity physical activity in a parallel design. After 34 months, a subgroup of 40 participants from the PREVIEW cohort [19] of Maastricht University in the Netherlands was recruited for the respiration chamber experiment [18]. Two participants of the 40 recruited dropped out due to a lack of time. Inclusion criteria for PREVIEW were previously described [19]. In short, participants were aged between 25 and 70 years and had to be overweight (BMI >25 kg/m^2^). Adults with prediabetes were included, defined as a fasting plasma glucose concentration between 5.6 and 6.9 mmol/L and/or a 2 h plasma glucose between 7.8 and 11.0 mmol/L following an oral glucose tolerance test (OGTT) [19].

For the substudy, participants arrived fasted (from 10:00 p.m. the evening before) at the research facilities in the Metabolic Research Unit Maastricht. At baseline, fasted blood samples were drawn, and anthropometric measurements included weight and body composition (BOD POD, Life Measurement Inc., Concord, CA, USA). Vascular function markers were assessed prior to and after their 48-h stay in the respiration chamber (in the following the terms pre- and post-respiration chamber will be used to refer to these measurement time points). In the respiration chambers energy expenditure was assessed by means of a continuous O_2_ and CO_2_ concentration measurement. The open-circuit indirect calorimetry was possible due to the airtight set up of the rooms with controlled climate and continuous fresh air ventilation [20]. Participants received three standardized meals per day, either with moderate-protein (MP: 15/55/30% energy from protein/carbohydrate/fat, respectively) or high-protein (HP: 25/45/30% energy from protein/carbohydrate/fat, respectively) content, which were in accordance with participants’ assigned intervention group during the PREVIEW study. Physical activity intensity based on the intervention was evenly spread (*n* = 8 high intensity and *n* = 12 moderate intensity in the HP group and *n* = 8 high intensity and *n* = 10 moderate intensity in the MP group) for the respiration chamber study. Fat content and quality were comparable between the groups. At the start of the substudy, participants did not significantly differ in their habitual dietary protein intake. All meals were provided through the laboratory and the investigators to reach energy balance tailored to individual energy requirements [18].

### 2.2. Cardiometabolic Risk Markers

#### 2.2.1. Ambulatory and Office Blood Pressure

Ambulatory BP (AMBP) was measured at regular intervals during the 48 h in the respiration chamber with a portable BP monitor (Mobil-O-Graph^®^ NG; APC Cardiovascular, Hartford, UK) as explained by Joris and colleagues [21]. In short, AMBP was measured in 15 min intervals during daytime (7:30 a.m.–11:30 p.m.) and in 30 min intervals during nighttime (11:30 p.m.–7:30 a.m.). The first measurement was discarded from the analysis and mean values for systolic blood pressure (SBP) and diastolic blood pressure (DBP), heart rate (HR), mean arterial pressure (MAP), and pulse pressure (PP) were calculated for the total 48 h period, as well as for daytime (7:30 a.m.–11:30 p.m.) and nighttime (11:30 p.m.–7:30 a.m.) separately. Additionally, systolic and diastolic dipping during the night were calculated as described [21].

Office BP was measured (Microlife, Wildnau, Switzerland) in supine position. The mean SBP and DBP were assessed, while the mean arterial pressure (MAP) and pulse pressure (PP) were calculated afterwards using the following formulae: MAP = 1/3 × SBP + 2/3 × DBP and PP = SBP − DBP.

#### 2.2.2. Serum Lipids and Lipoproteins

Fasting blood samples were taken in tubes equipped for serum separation (Becton, Dickinson and Company, Franklin Lakes, NY, USA) by venipuncture from the antecubital vein at pre- and post- respiration chamber time points. Clotting was allowed for 30 min at room temperature before centrifugation (10 min, 1500× *g*, 4 °C). Aliquots were stored at −80 °C until analysis. Serum aliquots were used for the analysis of total cholesterol (TC) (CHOD-PAP method, Roche Diagnostics System, Mannheim, Germany), high-density lipoprotein (HDL) cholesterol (precipitation method followed by CHOD-PAP method; Roche Diagnostics System), and triacylglycerol (TAG; GPO Trinder; Sigma-Aldrich Corp., St. Louis, MO, USA). Low-density lipoprotein (LDL) cholesterol concentrations were calculated using the Friedewald formula [22].

### 2.3. Vascular Function Measurements

Vascular function measurements were performed in a fasted state at pre- and post-respiration chamber time points, in a quiet and darkened room. The room was temperature controlled at 24 °C. Measurements were performed in supine position after an acclimatization period of at least 15 min. Detailed information has been described before by Joris et al. [23]. In brief, endothelial function was measured by brachial artery flow-mediated vasodilation (FMD) by use of ultrasound echography (Sonos 5500, Hewlett-Packard, Palo Alto, CA, USA). Pulse wave analysis (PWA; cAIxHR75) and carotid-to-femoral pulse wave velocity (PWV_c–f_) measurements were performed in triplicate with a tonometer (SphygmoCor v9; AtCor Medical, West Ryde, Australia) to assess arterial stiffness, and retinal vascular images were obtained to determine microvascular calibers using a nonmydriatic retinal camera (Topcon TRC-NW-300; Topcon Co., Tokyo, Japan).

### 2.4. Endocannabinoids and Endocannabinoid-Related Compounds

Plasma from EDTA tubes (Becton, Dickinson and Company, Franklin Lakes, NY, USA) were used for the analysis of AEA, PEA, OEA, 2-AG, and PREG as described [18]. Vacutainers contained 1% phenylmethylsulfonyl fluoride (PMSF) solution (10 mg PMSF in 1 mL methanol) and 5% 1N hydrochloric acid at final concentration. EDTA tubes and syringes for the blood sampling were ice chilled. After centrifugation (10 min, 1500 g, 4 °C) plasma was aliquoted and snap frozen immediately. AEA, 2-AG, OEA, and PEA were quantified with mass spectral analyses 5LC-M/MS (TSQ Quantum Access triple quadrupole instrument; Thermo-Finnigan, San Jose, CA, USA) [9,24,25] while PREG was quantified with GC-MS/MS (gas chromatography-tandem mass spectrometer) XLS Ultra Thermo mass spectrometer (Thermo-Finnigan, San Jose, CA, USA) via an AS3000 II autosampler [26]. Blood samples for endocannabinoid analysis were drawn directly before and 60 min after all three meals with one additional sample at 120 min after dinner. 

### 2.5. Statistical Analyses

Sample size was powered based on the primary outcome energy expenditure [27]. SPSS version 25 was used for statistical analyses (SPSS for Macintosh; SPSS Inc., Chicago, IL, USA). Data are presented as means ± standard deviation (SD). Data were log-transformed if not normally distributed as tested by the Shapiro–Wilk test. The trapezoidal method was used to calculate the area under the curve (AUC) for endocannabinoids and related substances [28] for the whole day. Differences at baseline between groups were determined by ANOVA and the treatment effect was calculated by an ANCOVA where the baseline variable was used as a covariate. Partial correlations with body fat % as covariate were used to assess associations between cardiovascular parameters and endocannabinoids.

## 3. Results

### 3.1. Baseline Characteristics

Participant characteristics prior to the respiration chamber stay were previously described [18]. In brief, no differences in age, BMI, body composition, and C-reactive protein (CRP) were found between the MP and HP intervention groups prior to the respiration chamber experiment (Table S1).

### 3.2. Cardiometabolic Risk Markers

#### 3.2.1. Office and Ambulatory Blood Pressure

Mean 24 h, mean daytime, or mean nighttime ambulatory SBP, DBP, PP, and MAP were not different between intervention groups. However, 24 h and daytime HR were significantly higher in the HP group (both *p* < 0.05). No differences in nighttime SBP or DBP dipping were observed (Table 1). Similar results were observed for the office blood pressure measurements (Table S2).

#### 3.2.2. Serum Lipids and Lipoproteins

Effects of the high- and moderate-protein diet in the respiration chamber on metabolic risk markers are presented in Table 2. Total cholesterol and LDL cholesterol concentrations were comparable between intervention groups at pre- and post-respiration chamber measurements and did not change in any of the groups. Changes in HDL cholesterol concentrations were not different between the MP and HP intervention group. The total cholesterol/HDL cholesterol ratio was not different between groups (Table 2). As described before, changes in TAG were less pronounced in the HP group [18].

### 3.3. Endothelial Function, Arterial Stiffness, and Retinal Microvascular Structure

FMD, an important marker reflecting vascular endothelial function, was not different between the groups and was also similar comparing pre- and post-respiration chamber time points, as presented in Table 3. Similar results were observed for markers of arterial stiffness, the central augmentation index adjusted for heart rate (cAIxHR75), and for PWV_c-f_. Finally, retinal microvascular calibers and the arteriolar-to-venular ratio did not differ between treatment groups and did not change (Table 3).

### 3.4. Endocannabinoids and Endocannabinoid-Related Compounds

As previously described [18], plasma 2-AG concentrations increased after meals and were generally higher in the HP condition. AEA, OEA, PEA, and PREG decreased throughout the day and were not affected by dietary protein intake [18]. AEA, 2-AG, OEA, and PEA were all positively associated with body fat percentage [29].

**Figure 1:**
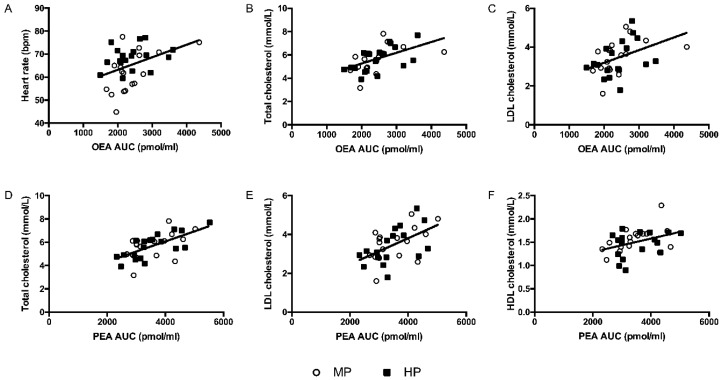
Association between OEA AUC with (**A**) daily heart rate (r = 0.373, *p* = 0.030), (**B**) baseline total cholesterol (r = 0.509, *p* = 0.002), (**C**) LDL cholesterol (r = 0.444, *p* = 0.010), and between PEA AUC with (**D**) total cholesterol (r = 0.594, *p* = 0.001), (**E**) LDL cholesterol (r = 0.527, *p* = 0.002), and (**F**) HDL cholesterol (r = 0.390, *p* = 0.025) in the whole group, as assessed with partial correlation analysis corrected for fat percentage. The regression line relates to the combined group. HP = high protein; MP = moderate protein; AUC = area under the curve; OEA = oleoylethanolamide; PEA = palmitoylethanolamide.

Plasma OEA concentrations were positively associated with 24 h and daily AMBP HR (Figure 1), total cholesterol, and LDL cholesterol (Figure 1), while PEA was positively associated with total cholesterol, LDL cholesterol, and HDL cholesterol (Figure 1). As there were no differences in PEA and OEA between the two intervention groups, the associations are presented for the whole group. In contrast, 2-AG, AEA, and PREG showed no associations with cardiometabolic risk or vascular function markers.

## 4. Discussion

In the current study the effects of a high-protein diet on cardiometabolic health and vascular function markers were examined in overweight participants. The study indicates that a diet with a higher protein content did not affect markers for metabolic health and vascular function, as serum lipoprotein concentrations and BP or markers for endothelial function, respectively. Only HR was higher with a HP diet. As a secondary aim, possible associations of markers for cardiometabolic health and vascular function with endocannabinoids and endocannabinoid-related compounds were examined. Here, the endocannabinoid-related compounds OEA and PEA were positively associated with cardiometabolic risk markers, such as serum cholesterol concentrations and HR, independent of protein intake.

In general, most of the individuals in the current study can be classified as hypertensive according to the 2018 European Society of Cardiology/ European Society of Hypertension (ESC/ESH) guidelines [30]. In a previous study, a HP diet reduced DBP compared to a normal protein diet, when combined with weight loss [31]. In the present study, no effects on BP were found comparing the two protein intervention groups. However, studies reporting effects of dietary protein on the regulation of blood pressure are conflicting [32,33], possibly due to the type of protein, whether it originates from plants or animals, or due to a specific amino acid composition. In the context of the amino acid composition, sulfur-containing amino acids like cysteine or methionine may raise blood pressure, while amino acids involved in gluconeogenesis may have lowering effects on blood pressure [34]. In the current study, a mixed-protein diet was used without any focus on the origin of protein or on a particular amino acid composition, which may explain the lack of effect on BP.

In contrast, HR was higher with a higher dietary protein content. This may be an explanation for the protein-induced effects on energy balance as discussed previously [27]. A positive association between HR and energy expenditure has been demonstrated and employed to develop a prediction model for energy expenditure in free living conditions based on heart rate [35].

The link between endocannabinoids and blood pressure is still unclear and clinical studies are limited. However, pharmacological blockade of the CB1 receptor reduced BP in humans [13,14], suggesting a possible relationship of the ECS with blood pressure. The fact that the relationship could not be confirmed in the present study may be explained by the different study set up, as previous studies focused on the deactivation of the receptor instead of investigating the relationship between BP and plasma endocannabinoid concentrations.

In our study, we observed a positive association between the endocannabinoid-related compound OEA with HR. A possible connection of cannabis use and HR was previously found, in which tetrahydrocannabinol (THC), an active compound of cannabis, increased HR without concomitant changes in BP [36]. As THC interacts to CB1, in contrast to OEA which acts CB1-independent, we suggest that effects on heart rate could also originate from a CB1 receptor independent mechanism.

Next to BP, metabolic risk markers were measured. No statistically significant differences in total cholesterol, LDL cholesterol, and HDL cholesterol concentrations were observed between the two protein intervention groups, possibly due to the short duration of the respiration chamber experiment. In addition, the total cholesterol/HDL cholesterol ratio did not differ. So far, the literature is inconclusive with regard to lipoprotein metabolism and protein intake. While beneficial effects on serum lipoprotein concentrations, independent of the amount of dietary fat intake, were only present in healthy, young individuals with a high-protein diet [37], studies in overweight and obese participants did not show a clear effect of a mixed-protein diet on serum lipoprotein concentrations [31,38,39]. In contrast, a meta-analysis investigating the effects of soy protein on lipoprotein concentrations indicated a reduction in LDL cholesterol [40,41], supporting the idea that the type of protein may be of great importance.

The ECS has been discussed to play a role in lipid metabolism [42], dyslipidemia, and lipogenesis [43]. In the current study, total cholesterol and LDL cholesterol concentrations were associated with OEA and PEA, independent of protein intake, suggesting a possible regulatory role for OEA and PEA in lipidemia. Meanwhile, 2-AG, AEA, and PREG were not related to any of the circulating risk markers.

Generally, OEA concentrations in saliva are higher in obese individuals compared to lean individuals [44] and associated with visceral fat content [45]. Animal data indicated a cholesterol lowering effect of OEA treatment [46]. While the animal data are not in agreement with the outcome of the current study, the ECS in animals may not necessarily be representative of the human ECS. PEA, in contrast, has been positively associated with total cholesterol and LDL cholesterol concentrations [47]. Interestingly, associations of PEA with HDL cholesterol were also positive in the current study whilst inverse associations were demonstrated previously [14]. The literature showed a positive association of 2-AG with TAG and a negative association with HDL cholesterol [9], while no associations were found in the current study.

No effects of a diet with a higher protein content were observed on vascular function which is in line with previous literature [48]. With regard to vascular function, most of the studies focused on the effect of specific proteins like whey or dairy products instead of a whole-protein approach. Whey protein intake was shown to improve FMD [49], whereas there was no effect of dairy intake on FMD and other markers for endothelial function [50,51], and no effect was reported by a mixed-protein approach [48]. Arterial stiffness improved after soy [52] and whey interventions [53]. However, due to the fact that this substudy was powered on energy expenditure, we cannot exclude power issues here as well. Literature regarding the relationship of vascular function and endocannabinoids is vague and barely investigated in clinical trials. In the current study, vascular functional markers did not relate to any of the endocannabinoid measurements. Similarly, OEA and PEA were not related to endothelial function in previous studies [54]. However, a connection between the ECS and vascular function cannot be ruled out completely as PEA was proposed to be a potential biomarker predicting coronary dysfunction in morbidly obese patients [54].

A limitation of the current study is the investigation of a diet with a higher protein/carbohydrate ratio compared to a diet with a lower protein/carbohydrate ratio, which omits the possibility of a clear separation between effects of an isolated higher protein concentration, a lower carbohydrate concentration, or a combination of both. In addition, the duration of the substudy was very short which makes a comparison with longer-term interventions difficult and we cannot rule out possible effects of the weight-loss intervention the participants followed before this substudy. Due to the complexity of protein quality and other factors of influence, follow-up studies are needed to confirm the relevance for ‘normal life’ conditions and to show whether the results can be extrapolated to overweight participants in general. The ECS is an extensive system, including different receptors, receptor variants, and ligands [11], that is associated with several anthropometric factors such as age and BMI [44,55], which further complicate interpretation of possible effects.

## 5. Conclusions

In conclusion, our data suggest that a diet with a higher dietary protein content did not affect cardiometabolic health and vascular function markers in overweight participants. Our data also indicate a possible relation between OEA and PEA and serum lipoprotein concentrations, independent of protein intake. Further research is needed to clarify the link between endocannabinoids, their related compounds, and cardiometabolic health from a more mechanistic perspective.

## Figures and Tables

**Table 1:** Ambulatory blood pressure measurements during 48 h of the respiration chamber stay with a moderate- or high-protein dietary diet.

		Moderate Protein (*n* = 18)	High Protein (*n* = 20)
24 h	SBP (mmHg)	133 ± 12	131 ± 12
	DBP (mmHg)	82 ± 8	79 ± 7
	MAP (mmHg)	106 ± 9	103 ± 9
	PP (mmHg)	51 ± 9	52 ± 9
	HR (bpm)	62 ± 9	67 ± 5 *
Daytime	SBP (mmHg)	139 ± 13	136 ± 13
	DBP (mmHg)	86 ± 9	83 ± 8
	MAP (mmHg)	111 ± 10	107 ± 9
	PP (mmHg)	53 ± 10	53 ± 9
	HR (bpm)	63 ± 9	69 ± 5 *
Nighttime	SBP (mmHg)	116 ± 13	117 ± 15
	DBP (mmHg)	68 ± 6	67 ± 8
	MAP (mmHg)	90 ± 9	90 ± 11
	PP (mmHg)	48 ± 10	48 ± 7
	HR (bpm)	58 ± 8	61 ± 7
Dipping	SBP (%)	16 ± 9	16 ± 3
	DBP (%)	21 ± 6	19 ± 7

* *p* < 0.05. Values are presented as mean ± SD. A one-way ANOVA was used to calculate differences between the groups. SBP = systolic blood pressure; DBP = diastolic blood pressure; MAP = mean arterial pressure; PP = pulse pressure; HR = heart rate; bpm = beats per minute.

**Table 2:** Metabolic risk markers at pre- and post-respiration chamber time points with a moderate- and high-protein diet.

	Moderate Protein (*n* = 18)		High Protein (*n* = 20)		Treatment Effect
	Pre ^1^	Post ^1^	Pre ^1^	Post ^1^	Difference in Change ^2^
Total cholesterol (mmol/L)	5.6 ± 1.1	5.6 ± 1.0	5.6 ± 1.0	5.5 ± 1.0	−0.1 (−0.3; 0.2)
HDL cholesterol (mmol/L)	1.4 ± 0.3	1.3 ± 0.2	1.5 ± 0.3	1.4 ± 0.3	0.0 (−0.1; 0.1)
LDL cholesterol (mmol/L)	3.5 ± 0.9	3.4 ± 0.8	3.4 ± 0.9	3.3 ± 0.8	0.0 (−0.2; 0.2)
Total cholesterol/HDL cholesterol ratio	4.0 ± 0.9	4.4 ± 1.1	3.6 ± 0.7	3.9 ± 0.7	−0.1 (−0.3; 0.1)

^1^ Values are means ± SD; ^2^ Treatment effects (95% confidence interval (CI)) were obtained from a one-factor ANCOVA with baseline value as covariate. HDL = high-density lipoprotein; LDL = low-density lipoprotein.

**Table 3:** Vascular function measurements at pre- and post-respiration chamber time points with a moderate- or high-protein dietary diet.

	Moderate Protein (*n* = 18)		High Protein (*n* = 20)		Treatment Effect
	Pre ^1^	Post ^1^	Pre ^1^	Post ^1^	Difference in Change ^2^
**Vascular function**					
Baseline brachial diameter (cm)	0.58 ± 0.14	0.58 ± 0.10	0.57 ± 0.12	0.56 ± 0.14	−0.02 (−0.05; 0.01)
FMD (%)	3.4 ± 2.1	3.6 ± 2.3	4.4 ± 3.2	4.4 ± 3.3	0.4 (−1.5; 2.3)
**Arterial stiffness**					
PWV_c–f_ (m/s) ^3^	8.8 ± 1.5	8.6 ± 1.1	8.8 ± 1.3	8.8 ± 1.8	0.2 (−0.6; 0.9)
cAIxHR75 (%)	22.1 ± 8.2	21.5 ± 7.1	25.4 ± 7.0	23.3 ± 8.8	−0.7 (−4.6; 3.2)
**Retinal microvascular structure**					
Arteriolar width (μm) ^4^	128 ± 19	127 ± 20	124 ± 23	125 ± 19	2.4 (−1.9; 6.8)
Venular width (μm) ^4^	222 ± 25	220 ± 24	214 ± 29	215 ± 29	2.2 (−2.1; 6.5)
Arteriolar-to-venular ratio ^2^	0.58 ± 0.09	0.58 ± 0.09	0.58 ± 0.1	0.59 ± 0.08	0.01 (−0.02; 0.04)

^1^ Values are presented as means ± SD; ^2^ Treatment effects are mean changes (95% CI) obtained from one-factor ANCOVA with baseline value as covariate. ^3^ Moderate-protein group (MP): *n* = 17; high-protein group (HP): *n* = 19. ^4^ MP: *n* = 17; HP: *n* = 18. FMD = flow-mediated dilation; PWV_c–f_ = carotid-to-femoral pulse wave velocity; cAIxHR75 = central augmentation index adjusted for heart rate, derived from pulse wave analysis (PWA) measurement.

## Supplementary Materials

The following are available online at https://www.mdpi.com/2072-6643/12/5/1512/s1, Table S1: Subject characteristics of the moderate- and high-protein group prior to the respiration chamber stay. Table S2: Office blood pressure at pre- and post-respiration chamber time points with a moderate- or high-protein diet.
