# Supplementary material for: Effects of a High-Protein Diet on Cardiometabolic Health, Vascular Function, and Endocannabinoids—A PREVIEW Study
Source: Nutrients. 2020 May 22;12(5):1512. doi: 10.3390/nu12051512 (PMC7284520; doi:10.3390/nu12051512)
Supplement: Supplementary file 1 [file nutrients-12-01512-s001.pdf]

Table S1. Subject characteristics of the moderate- and high-protein group at prior to the respiration chamber stay

	Moderate protein	High protein
N (f/m)	18 (9/9)	20 (13/7)
Age (year)	65.1 ± 5.8	64.0 ± 6.2
BMI (kg/m²)	29.0 ± 3.8	28.9 ± 4.0
Body-fat (%)	39.5 ± 8.1	40.7 ± 7.7
Fat mass (kg)	33.9 ± 7.1	34.8 ± 8.8
Fat-free mass (kg)	52.5 ± 10.9	50.8 ± 11.3

Values are means ± SD. BMI = Body Mass Index.

Table S2. Office blood pressure at pre- and post-respiration chamber time points with a moderate- or high-protein diet

	Moderate protein (n=18)		High protein (n=20)		Treatment effect
	Pre[1]	Post[1]	Pre[1]	Post[1]	Difference in change[2]
Brachial SBP (mmHg)	126±10	127±10	130±11	127±12	-2 [-8; 3]
Brachial DBP (mmHg)	77±7	77±6	78±6	76±7	-2 [-5; 2]
Brachial MAP (mmHg)	93±7	93±6	95±7	93±8	-2 [-5; 2]
Brachial PP (mmHg)	49±7	50±10	52±10	51±9	-1 [-5; 4]
HR (bpm)	62±9	57±6	65±7	62±6	3 [0; 6]

[1]Values are means±SDs. [2] treatment effects [95% CI] were obtained from a one-factor ANCOVA with baseline value as covariate. BP = blood pressure; SBP = systolic blood pressure; DBP = diastolic blood pressure; HR = heart rate; bpm.: beats per minute.
